# High plasma homocysteine and insulin resistance in patients with polycystic ovarian syndrome 

**Published:** 2011

**Authors:** Tayebe Hemati, Nasrin Moghadami-Tabrizi, Fateme Davari-Tanha, Bahram Salmanian, Pouya Javadian

**Affiliations:** 1 Department of Obstetrics and Gynecology, Mirza Kouchak Khan Hospital, Tehran University of Medical Sciences, Tehran, Iran.; 2 School of Medicine, Tehran University of Medical Sciences, Tehran, Iran.

**Keywords:** *PCOS*, *Homocysteine*, *Insulin resistance*, *Infertility*

## Abstract

**Background:**

Polycystic ovarian syndrome (PCOS) is a common disease among women in fertility ages and cause severe insulin resistance. Hyperhomocysteinaemia is said to be among the features of PCOS that could influence its outcome.

**Objective:**

This study aimed to investigate whether hyperhomocysteinaemia exists in PCOS and if it is related to insulin resistance in the affected patients.

**Materials and Methods:**

This prospective study was carried out in a university based fertility clinic. Sixty four PCOS patients and 50 normo ovulatory controls were reviewed for fasting glucose, insulin, homocysteine, luteinizing hormone (LH), and follicle-stimulating hormone (FSH) plasma levels in the blood sample of the 3^rd^ day of their menstrual cycle. Insulin resistance was determined with the fasting glucose (mmol/L) to insulin (mIU/L) ratio and HOMA-IR (Homeostasis model assessment-Insulin resistance). Independent-samples T-test and linear regression test were utilized to analyze the obtained data.

**Results:**

Homocysteine levels compared between PCOS patients and control group showed a significant difference. PCOS group was divided into insulin resistant (IR) (LogHOMA-IR≥0.57) and non insulin resistant (NIR) patients. The IR group had significantly higher homocysteine (p-value=0.02), fasting insulin and glucose levels (p-value<0.001) rather than NIR group.

**Conclusion:**

PCOS patients have a leaning toward hyperhomocysteinaemia and insulin resistance. Insulin resistant patients are found to have higher homocysteine level.

## Introduction

Polycystic ovarian syndrome (PCOS) is a common disease among women in fertility age. It causes ovulation disturbances, hyper androgenism, infertility and increased abortion rate. Obesity, hyper insulinemia, diabetes, hypertension, dyslipidemia, atherosclerosis and vascular diseases are other problems ascribed to PCOS (1-5). It has been shown that PCOS can cause severe insulin resistance and its secretion disturbances to some extent.Homocysteine is an intermediate substance in methionine metabolism. An increased homocysteine plasma level classically happens due to an enzymatic defect in the aforementioned process. 

 The recent studies have shown there are so many non-enzymatic factors affecting homocysteine levels as well (6-8). According to the insulin role in inhibition of hepatic cystathione beta synthase, which is an enzyme involved in methionine metabolism, insulin plasma level is also introduced as a determining factor of homocysteine levels (9, 10). Insulin resistance seems to increase the homocysteine levels (6, 8, 11). 

There are several studies regarding the role of homocysteine in pathogenesis of vascular diseases. Homocysteine is found to be an effectual factor in coronary artery diseases (CAD). Homocysteine level higher than 11 µmol/L is detected in 30% of CAD patients and increases the mortality rate by 3 times (12, 13). Herein, we aim to evaluate the relation between homocysteine level and PCOS, and its association with insulin resistance that could affect the outcome of PCOS short and long term management.

## Materials and methods

Sixty four patients referring to the infertility unit at a university based fertility clinic, affiliated to Tehran University of Medical Sciences, were selected to be studied in a prospective study, in 2008-2009. The diagnosis of PCOS was made according to the Rotterdam European Society of Human Reproduction and embryology (ESHRE)/ American Society for Reproductive Medicine (ASRM), sponsored PCOS consensus workshop group guide lines (14). Those criteria are well accepted for PCOS diagnosis (1). The study and its methodology were approved by ethic committee of Tehran University of Medical Sciences. All possible risks were explained to patients and informed consent was obtained from all participants. 

None of the patients were diagnosed with diabetes or had any smoking habits. Those who received metformin, folic acid or phenytoin were also excluded.

Fifty other patients referring to the same unit who had non ovarian problems were randomly selected as the control group utilizing a pseudo-random number generator. They had normal ovarian and menstrual cycle; who did not present with any of clinical and ultrasound signs and symptoms of PCOS. They were matched with the PCOS group regarding age and physical activity to omit any known confounding factor.

**Laboratory tests**

All cases in both groups were weighed in two different days and their height was measured. BMI was calculated by dividing the weight (kg) by squared height (m^2^). The basic plasma level of LH, FSH and fasting glucose were measured in the 3^rd^ day of a spontaneous or progesterone induced menstrual cycle before any treatments. Fasting insulin and homocysteine total plasma levels were also measured in the same day using Eliza enzyme immunosorbent assay (Calbiotech Inc., CA). Serum prolactin, thyroid hormone and 17-hydroxy progesterone along with BUN and creatinine were measured. Those were inspected not to exceed the normal levels.

Insulin resistance is determined with several indices, including: fasting insulin total plasma level (mIU/L), the fasting glucose (mmol/L) to insulin (mIU/L) ratio, and HOMA-IR (Homeostasis model assessment-Insulin resistance) calculated by Insulin (mIU/L) * glucose (mmol/L)/ 22.5. 

Fasting insulin total plasma level and fasting glucose to insulin ratio have more restrictions rather than HOMA-IR, are less sensitive physiologically, and are not useful in abnormal glucose levels. Therefore, we utilized the HOMA-IR in the rest of this study. We converted HOMA-IR into logarithmic scale, since it rarely has normal distribution (15, 16). Insulin resistance was inspected also by measuring the insulin levels 2 hours after glucose loading. 150 pmol/ L was considered the cut-off.

To determine insulin resistance regarding each index, the upper limit of the normal ranges was considered mean±2SD of the control group. 

**Statistical analysis**

We used SPSS software version 16 (SPSS Inc. Chicago, IL, USA) for statistical analysis. p-value of 0.05 and lower was considered significant. Independent-samples T-test was utilized to compare differences between parametric data groups. In order to express the conditional distribution of bivariate correlated items linear regression test was done.

## Results

Homocysteine levels compared between PCOS patients and control group showed a significant difference, 10.96±7.27 µmol/L in all PCOS vs. 6.8±1.95 µmol/L in controls (p-value <0.001). The clinical and biochemical data of all patients regarding their differences is shown in Table I.

Ninety five percent (Mean+2SD) of the control group had homocysteine levels lower than 10.7µmol/L, while the normal range of homocysteine is usually considered between 5 to 11 µmol/L (3). 35.94% of cases (23/64) in the PCOS group had homocysteine levels higher than 10.7 µmol/L (p<0.001), who were regarded hyperhomocysteinemic.

Insulin resistance determined with different indices in our study. For each variable, the upper limit was defined as mean±2SD of the normal control group. Therefore 20 mIU/L, 0.3, and 0.57 were regarded as the cut-off levels for fasting insulin level, glucose to insulin ratio, and Log HOMA-IR respectively. 

In the same order, 35.94%, 48.44% and 57.81% of the patients had insulin resistance. Measuring insulin level 2 hours after glucose loading, 45.31% were resistant. All the aforementioned results were found to be significantly different compared with those of the control group (p<0.001, all groups). Log HOMA-IR was utilized in the rest of the study. 

PCOS group was divided into insulin resistant (IR) (Log HOMA-IR ≥ 0.57) and non insulin resistant (NIR) (Log HOMA-IR < 0.57) patients. The IR group had significantly higher homocysteine (p-value=0.02), fasting insulin and glucose levels (p-value<0.001) rather than NIR group. 

However, no association was found between Log HOMA- IR and BMI (p-value=0.18) (Table II). 18.52% (5/27) of NIR-PCOS patients had increased levels of homocysteine (>10.7 µmol/L), while 48.65% (18/37) of IR-PCOS were found hyperhomocysteinemic (Homocysteine in IR-PCOS 12.6±7.9 Vs. Homocysteine in NIR-PCOS 8.6±5.6, p-value= 0.02).

More number of PCOS group showed BMI≥ 23 in comparison with the control group (p< 0.06), but the higher BMI did not have any significant association neither with homocysteine nor with insulin resistance in PCOS group, Homocysteine and Log HOMA-IR 12.3±6.9 and 0.58±0.27 in BMI ≥ 27 PCOS group respectively vs. 10.5±7.3 and 0.58±0.24 in BMI<27 PCOS group (p-value= 0.4 and 0.9). 12.88% of the PCOS group had BMI ≥ 27 Kg/m^2^.

Increased LH to FSH ratio was significantly found in PCOS patients rather than the control group (p<0.001), whether they were obese or insulin resistant or none. Age was only related to BMI (p-value= 0.001) but with no other variable.

All the insulin resistance indices were correlated with homocysteine levels. (Fasting insulin r= 0.513 p< 0.001, Glu/ Ins r=-0.3 p= 0.015, Log HOMA- IR r= 0.3 p= 0.015, Ins2hr r= 0.37 p= 0.002). 

**Figure 1:**
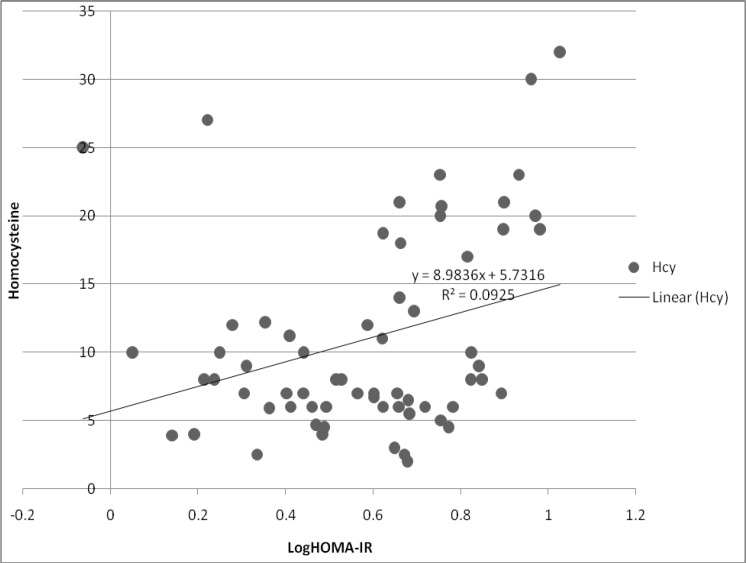
Linear regression between homocysteine levels and logarithmic transformation of HOMA-IR

The correlation between homocysteine levels and logarithmic transformation of HOMA-IR, ANOVA linear regression test, is shown in figure 1. 

**Table I:** Clinical and biochemical data of all patients

	**Control (N=50)**	**PCOS (N=64)**	**p-value**
Age (years)	29.1±3.2	31.09±8.9	0.08
BMI (kg/m^2^)	22.1±0.25	22.7±3.2	<0.06
Homocysteine (µmol/l)	6.8±1.95	10.9±7.2	<0.001
Insulin (mIU/l)	10.6±4.7	18.9±8.6	<0.001
LH/FSH	0.8±0.2	1.77±1	<0.001
Glucose/Insulin ratio	0.56±0.13	0.32±0.14	<0.001
Log HOMA-IR	0.23±0.17	0.58±0.24	<0.001

PCOS: polycystic ovarian syndrome, BMI: body mass index, Log HOMA-IR: logarithmic transformation of homeostasis index of insulin resistance.

**Table II:** Variables and their differences in insulin resistant and non insulin resistant PCOS patients

	**Number**	**Homocysteine**	**Fasting glucose**	**Fasting insulin**	**Glucose/Insulin**	**BMI**
PCOS-NIR	27	8.6±5.6	83.9±11	11.1±2.7	0.44±0.12	25.3±2.9
PCOS-IR	37	12.6±7.9	97.9±11.2	24.6±6.8	0.23±0.07	24.2±3.4
p value		0.02	<0.001	<0.001	<0.001	0.18

Values are mean±SD.

PCOS: polycystic ovarian syndrome, NIR: non insulin resistant, IR: insulin resistant.

## Discussion

There were significant differences found in homocysteine levels and insulin resistance between the PCOS patients and the control group. PCOS group stratified into insulin resistant and non-insulin resistant patients. Thereby, significant difference was found between homocysteine levels which propose significant correlation between insulin resistance and homocysteine.

Recently, the local or systemic effects of insulin resistance have been studied. Evidences provided have shown that hyperinsulinemia and some phenotypes of insulin resistance syndrome could have several metabolic effects in general population, including hyperhomocysteinemia (17). 

Some previous studies had shown the relation between increased homocysteine levels and insulin resistance in particular groups of fertile women. Laivuori *et al* investigated the association of insulin resistance and hyperhomocysteinaemia in preeclamptic pregnant women (18). They found that such a relation exists in preeclampsia, but not in the control group.

According to our findings insulin resistance is higher in the PCOS patients. They have a leaning toward hyper homocysteinaemia as well. However, homocysteine levels were found to be significantly higher in IR group than the NIR (p=0.026). That could suggest the insulin resistance role in hyperhomocysteinaemia, but not as the only factor. Such increase could be due to relative hyperandrogenism or steroidal sex hormones influence in PCOS (19). 

Bayraktar *et al* have shown a relation between homocysteine plasma levels, insulin resistance and androgen levels in PCOS. Such a relation is not found in congenital adrenal hyperplasia patients (20). 

However, there was an investigation that could not find any connection between polycystic ovaries and insulin levels (21). The only criterion considered by that study was ultrasound features. None of the other PCOS criteria was taken into consideration. That could give a reason to their contradictory findings. Another study suggested that hyperhomocysteinaemia in PCOS is independent of insulin resistance and is due to other factors. Their results may be affected by their small sample size (n=29) (22). 

Rosolová *et al* found an unexpected reverse relation between insulin resistance and homocysteine level in healthy people (23). To our opinion, such controversial results are caused by the difference in PCOS, insulin resistance and hyperhomocysteinaemia definition among populations studied.

As shown in several researches, homocysteine is in a positive relation with the risk of cardiovascular diseases (12, 13). Implantation disturbances and early pregnancy losses are both common in PCOS, even after the correction of ovulation disturbances and increased LH levels or hyperandrogenism. All those could be in association with hyper homocysteinaemia due to its effect on vascular structure. 

Insulin resistance is independently a risk factor for cardiovascular diseases, diabetes, nephropathy due to hypertension, and dyslipidemia (24). All those which are related to the metabolic syndrome are intensified by hyperhomocysteinaemia. Then, PCOS could be considered as a type of insulin resistance syndrome or as an early sign of that syndrome. However, it should be kept in mind that the homocysteine plasma level is affected by several factors.

If the PCOS is diagnosed, it’s better to have in mind the other metabolic complications as well. Gynecologists should consider both the short term (infertility) and long term (cardiovascular and metabolic diseases) outcomes in their management. Further studies are needed to investigate the effect of insulin sensitizers on homocysteine plasma level. 

Regarding the result of this study, approving hyperhomocysteinaemia and its relation to insulin resistance, and vascular abnormalities caused by them shown in other studies, it seems that clinical examination concerning those complications is a matter of importance in PCOS management. Active treatment of hyperhomocysteinaemia may decrease PCOS morbidity. Prospective studies could objectively show the promising role of such treatments in PCOS.
